# Frequency and short-term persistence of haematuria and/or proteinuria in neonates: a cohort study

**DOI:** 10.1007/s00431-026-06859-w

**Published:** 2026-03-26

**Authors:** Jochen Kittel, Christine Seilbeck, Susanne Brandstetter, Michael Kabesch, Michael Melter, Angela Köninger, Christian Apfelbacher, Andreas Ambrosch, Tobias Geis

**Affiliations:** 1 University of Regensburg, Children’s Hospital (KUNO), Hospital St. Hedwig of the Order of St. John, Regensburg, Germany; 2 Present Address: Research and Development Campus Regensburg (WECARE), Hospital St. Hedwig of the Order of St. John, Regensburg, Germany; 3 University Clinic of Gynecology and Obstetrics, Hospital St. Hedwig of the Order of St. John, Regensburg, Germany; 4 Institute of Social Medicine and Health Systems Research, Otto Von Guericke University Magdeburg, Magdeburg, Germany; 5 Institute for Laboratory medicine and Microbiology, Hospital of the Order of St. John, Regensburg, Germany

**Keywords:** Birth cohort, Haematuria, Proteinuria, Newborn, Kidney disease, Urine test

## Abstract

**Purpose:**

Haematuria and proteinuria may point to kidney diseases, but may also be found incidentally. Among schoolchildren, many studies suggest their prevalence to around 1%. In neonates, the frequency and persistence of haematuria and/or proteinuria in the general population have so far not been investigated systematically.

**Methods:**

In the course of the prospective KUNO-Kids Health Study, urine samples were collected and analysed by dipstick in asymptomatic neonates on days three to five after birth. Those with positive findings underwent a maximum of two follow-ups until 16 weeks of age and factors associated with haematuria and/or proteinuria were explored.

**Results:**

Of 509 participants with a urine sample available, 27% (*n* = 139) exhibited positive results. Of these, 58% (*n* = 81) had isolated haematuria, 21% (*n* = 29) had isolated proteinuria, and 21% (*n* = 29) had both. Of all children with positive urine tests, 76% (*n* = 105) underwent a first follow-up (mean 7 weeks later), and only in 1.9% (*n* = 2) was a positive result found. In the second follow-up (mean 2 weeks later), no positive results were detected anymore. Positive urine test results were more common in females and after vaginal delivery (*p* < 0.001 and *p* = 0.037, respectively).

*Conclusion*: The present study identified haematuria and/or proteinuria in a significant proportion of healthy newborns shortly after birth, but results returned to normal within weeks in all participants available for follow- up. Thus, isolated findings of haematuria and/or proteinuria in newborns should be interpreted with caution.

**Table:** 

**What is known:** *• Previous studies have reported the prevalence and persistence of haematuria and/or proteinuria in school- age children, but data on haematuria and/or proteinuria in neonates are scarce.*	
**What is new:** *• Positive urinary test results for haematuria and/or proteinuria are common after birth and were found in 27%.* *• Positive urinary test results returned to normal within 16 weeks in all children tested.*

**Supplementary Information:**

The online version contains supplementary material available at 10.1007/s00431-026-06859-w.

## Introduction

A wide range of renal diseases can result in early renal insufficiency. Early screening for indicators to detect such renal diseases and to avoid permanent damage would be desirable. The presence of haematuria and/or proteinuria could be such an early indicator [1].

As part of the German paediatric check-up programme for the early detection of degenerative kidney disease, urine screening for haematuria and/or proteinuria is carried out for the first time at age 4 years [2].

Basic urine testing, most often applied in paediatric practice to detect urinary tract infections, is performed using rapid dipstick tests or photometry [1–5]. It is not uncommon for haematuria and/or proteinuria to be detected by chance, and their clinical relevance is often unclear in such cases [6–8]. In clinical paediatric practice, the presence of haematuria and/or proteinuria is usually considered as temporary, and only rarely as a long-term problem [4–9].

While numerous studies have assessed haematuria and/or proteinuria in children, there is little data on its applicability and relevance in the neonatal period [10, 11]. No studies combining initial urine screening at birth with 24- week follow-up assessments have been conducted to date. Knowledge on this topic could help to determine the feasibility and usefulness of urinary testing in healthy neonates as an early screening tool in the future.

## Patients and methods

### Study cohort

The present study was based on the KUNO-Kids Health Study (KKHS), a prospective birth cohort sampled from the general population described in detail elsewhere [12]. A total of 1983 participants from KKHS, who were recruited between July 2015 and September 2017, were eligible to participate in this study. Only asymptomatic neonates (born at > 29 gestational weeks) without any clinical evidence of kidney disease (such as oedema) were included. None of the participating children had undergone bladder catheterisation prior to the urine test.

Baseline screening with follow-up of positive urine findings was performed. Initial urine testing was performed between days three and five after birth. KKHS data collected until the age of 24 weeks were used in this analysis, and demographic data of the cohort selection are presented in Table 1.

**Table 1:** Characteristics of the 509 infants included in the screening and of the remaining 1,473 cases. Individual missing data for sex, gestational age and birth weight. Note: Continuous data are reported as mean and standard deviation and categorical data as absolute numbers and percentages. P-value* for differences between the study population with and without a urine sample. Chi^2^ test for categorical variables, t-test for metric variables

	Study population with urine sample for analysis (*n* = 509)	Study population without urine sample (*n* = 1473)	p- value*
Sex	334 (65%) males 174 (34%) female	667 (46%) males 806 (54%) female	< 0.001
Gestational age, weeks	40 (SD 1.58; range 30.0–43.0)	40 (SD 1.60; range 26.0–43.0)	0.204
Birth weight, grams	3354 (SD 541; 1780–4790)	3344 (SD 482; 1800–4760)	0.706
Delivery mode: cesarean section	168 (33%)	379 (26%)	0.002

### Urine testing

To collect urine samples for this study, non-sterile disposable urine bags ® (PZN (Pharmazentralnummer): 07485590 from Dahlhausen, Germany) were applied by a trained nurse following cleansing of the external genitalia with either Prontosan® or sterile water and drying. If no urine could be collected on the first attempt of the initial screening, no second attempt was performed.

Urine samples were evaluated photometrically using Combur-Test® (PZN: 04659339 from Roche Diagnostics, Germany), a rapid dipstick urine test system, to assess leukocyturia, nitrite, pH value, protein, glucose, ketones, urobilinogen, bilirubin, and blood within a maximum of 24 h after collection. If the amount of urine was less than 2 ml, only a visual reading of the dipstick test was conducted.

Dipstick results for photometric or visual readings are displayed in five categories: negative, (+), +, + +, + + +. Results of +, + + or + + + were considered positive. Trace (+) results were classified as negative.

Due to the limited number of qualified available technical assistants, light microscopy could not be performed to verify the Combur-Test® tests results in this study.

If haematuria and/or proteinuria were detected in the initial test on the third to fifth day after birth, probands and their parents were invited to a first follow-up examination within 4–12 weeks after birth. Once again, urine was collected using a urine bag, and the sample was analysed as described above in the hospital laboratory. For cases with a negative test result, no further follow-up was scheduled. For children with a positive urinary test result for blood or protein, a second follow-up examination was planned within 2 weeks.

#### Data management and statistical analysis

Haematuria and/or proteinuria values were entered into an electronic case report form. Subsequently, the data were merged with the interview and questionnaire data from KKHS. Manually entered data were controlled by a second person; potentially implausible findings were checked and discussed on a case-by-case basis if necessary. Cases with missing values were not excluded from the data set, no statistical imputation of missing values was performed. We assessed the normality of continuous variables using Q-Q plots and calculated descriptive statistics. Chi^2^-tests and t-tests for independent samples were used to compare the characteristics of children included in the analytical samples with those of children not included. Chi^2^-tests were performed to determine whether child-related variables, such as mode of delivery, febrile infection in the personal history or family history, were associated with haematuria and/or proteinuria. All analyses were computed using IBM© SPSS© Statistics Version 23.0.

## Results

In 509/1983 newborns of the study cohort, an initial urine sample could be obtained (26%). As shown in Table 1, urine collection was more successful in boys than in girls (65% vs. 34%).

**Fig. 1:**
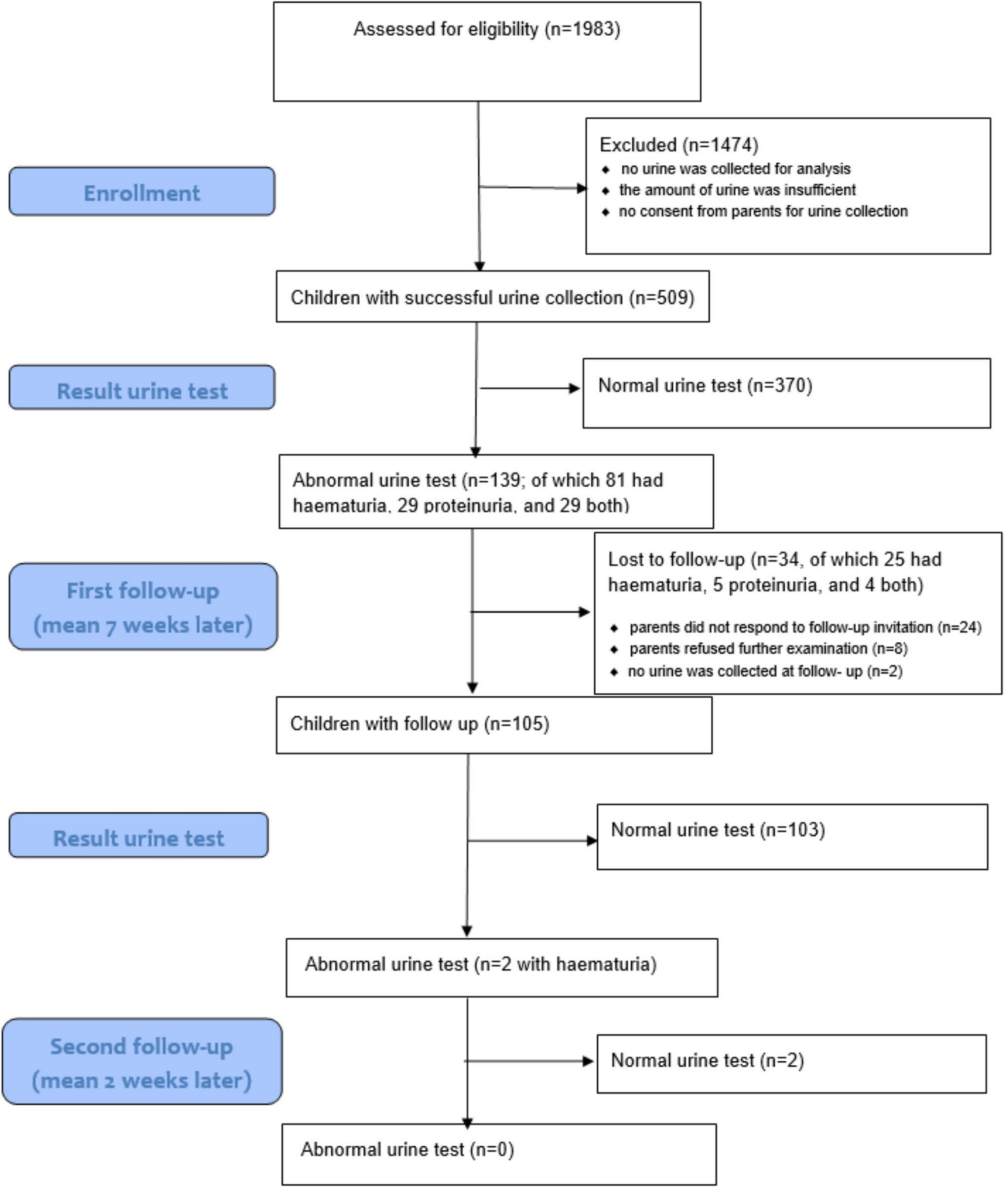
Flowchart diagram

Of the 509 children of whom urine samples were successfully collected, 27% (*n* = 139) had positive results (see Fig. 1) for haematuria and/or proteinuria. Of these, 58% (*n* = 81) had isolated haematuria, 21% (*n* = 29) had isolated proteinuria, and 21% (*n* = 29) had combined haematuria and proteinuria (see Table 2 for details). In addition, no case showed macrohaematuria.

**Table 2:** Cross-tabulation of 139 cases with haematuria (+, + + or + + +) and/or proteinuria (+, + + or + + +) on day 3–5; *n* = cases (% = percent). Percentages refer to total sample size of 139 cases with haematuria and/or proteinuria

	No haematuria *n* (%)	Haematuria + *n* (%)	Haematuria + + *n* (%)	Haematuria + + + *n* (%)	Total *n* (%)
No proteinuria n (%)		**55 (39.5)**	**14 (10.1)**	**12 (8.6)**	**81 (58.3)**
Proteinuria + n (%)	**29 (20.9)**	**22 (15.8)**	**2 (1.4)**	**4 (2.9)**	**57 (41)**
Proteinuria + + n (%)	**0**	**0**	**1 (0.7)**	**0**	**1 (0.7)**
Proteinuria + + + n (%)	**0**	**0**	**0**	**0**	**0**
Total n (%)	**29 (20.9)**	**77 (55.4)**	**17 (12.2)**	**16 (11.5)**	**139 (100)**

Of the 139 infants with positive baseline findings, 105 (75.5%) returned for first follow-up. Among these 105 infants, 81 (77.1%) were evaluated at the hospital and 24 (22.9%) in a paediatric office. Of the 105 infants assessed, 2 (1.9%) had a persistent positive result. 103 (98%) children had urine tests free of proteinuria or haematuria. The two children with twice a positive result, finally showed normal urine tests at the second follow-up examination.

Initially positive urine test results were found in 40% (*n* = 69) of girls and 21% (*n* = 70) of boys. Thus, female newborns showed significantly more often combined haematuria and/or proteinuria (χ^2^(1) = 20.122, *p* < 0.001). Furthermore, newborns delivered vaginally (30%) also showed a higher frequency of a positive urine test result compared to newborns delivered by a cesarean section (21%) (χ^2^ (1) = 4.367, *p* = 0.037). There was no significant association between positive test results and gestational age at birth (preterm/term), birth weight, or multiple/singleton birth. In 16% (*n* = 70) of the children with initially successfully collected urine, a familiy history of kidney disease or urological disease in the family was reported (data available for 442 of 509 families). Out of these, 20% (*n* = 14) had a positive urine result. No statistically significant association was found between the frequency of positive neonatal urine tests and a family history of nephrological or urological disease (parents and grandparents, both maternal and paternal) (χ^2^(1) = 2.673, *p* = 0.102). In the first week of life, 1.7% (*n* = 6) of the children experienced a febrile infection, sepsis, or urinary tract infection (data available for 356 of 509 children). Of these children, 33% (*n* = 2) had a positive urine test. No association was identified between the presence of haematuria and/or proteinuria and subsequent febrile infections, sepsis, or urinary tract infections during the first week of life (χ^2^ (1) = 0.065, *p* = 0.798).

At the age of 24 weeks, 387 out of 509 questionnaires were received from parents whose children had initially urine test results. Based on parental questionnaires, 2.8% (*n* = 11/387) children were diagnosed with a urinary tract infection, kidney disease and/or urinary tract disease within the first 24 weeks of life. Of these children, only one had a positive urine result in the neonatal urine examination at the age of three to five days, no association was identified (χ^2^(1) = 2.080, *p* = 0.149).

## Discussion

Our study detected positive, but only transient, urine test results for haematuria and/or proteinuria in more than a quarter of newborns. During subsequent follow-ups over 16 weeks, these results returned to normal. Haematuria and/or proteinuria at birth were associated with female sex and vaginal mode of delivery.

Our study is, to the best of our knowledge, the first to test for the persistence of both haematuria and/or proteinuria not only early after birth, but also over a 6-month follow-up period. A historical study of macrohaematuria in newborns was conducted in the USA between 1950 and 1967, involving 35 patients [10]. Between 2007 and 2008, Falakaflaki et al. performed a urine strip test for haematuria on 400 healthy newborns in Iran [11] without long- term follow- up. Our findings revealed no association between the presence of haematuria and/or proteinuria in the initial days of life and urinary tract infections, urological diseases, or nephrological diseases within the first 24 weeks.

Previous studies suggested that persistent micro- and macrohaematuria are more prevalent among premature infants, especially those receiving care in a neonatal intensive care unit (NICU) [10]. We did not replicate these analyses, as children born before gestational week 30 or treated in the NICU were excluded from our study. However, in those children born beyond gestational week 30, no correlation between haematuria and/or proteinuria and gestational age was identified.

One potential hypothesis for the high prevalence of haematuria and/or proteinuria on days three to five, without any correlation to kidney disease, is urine contamination. Certain influencing factors can lead to false positives, and some are particularly relevant in the neonatal period. The genitals of newborns may still be heavily contaminated with maternal vaginal secretion including blood and proteins, potentially resulting in false positive test results, also for leukocytes [15, 16]. Thus, the genitals must be thoroughly cleansed prior to urine collection to enhance the accuracy of the urine dipstick analysis [15–19], a procedure applied in our study. The extent of contamination may also depend on the mode of delivery, and it is conceivable that more contamination may be found after vaginal delivery (compared to caesarean section). Additonally, the delivery of a baby by caesarean section normally is not accompanied with blood contamination whereas vagina birth is associated with fetal contact to maternal stool, vaginal smear and blood following microlesions of the maternal genital tract. Indeed, this was identified as a factor associated with haematuria and/or proteinuria in our study. Furthermore, using the urine bags may lead to contamination by stool. For the purpose of future studies, an alternative method for neonatal urine testing could be used, such as 'clean catch', after thorough cleaning of the genitalia.

Another potential hypothesis is that maternal oestrogen may contribute to transient urinary findings in female neonates. This is because oestrogen can occasionally result in short uterine bleeding episodes in newborns due to the oestrogen-responsive endometrium [20]. Further possible hypotheses for transient haematuria and/or proteinuria include foetal stress or healing processes after the trauma of birth, which may both result in inaccurate urine test results [21]. However, this has not yet been investigated systematically, neither by us nor by other studies.

Based on our results, it is questionable whether urine testing at birth could be feasible for screening for kidney diseases. A more suitable time could be between three and four weeks of age [17, 18]. However, it is important to note that screening at this age may pose new challenges, as newborns are not typically seen in hospitals at this point in life. They are more frequently seen by paediatricians in their offices (at least in Germany). That setting would require simple screening methods, but urine bags and dipstick tests have several limitations. Thus, a screening for kidney diseases soon after birth is still a long way from becoming a reality.

In daily clinical practice, findings of haematuria and/or proteinuria in healthy infants detected during the first days of life need to be followed up on and be interpreted with caution, as they are most likely incidental or false positives. There is a possibility of dipstick false positive results for blood due to hemoglobin or myoglobin, and of protein detection being influenced by urine concentration.

Overall, the study's findings have limited generalisability due to the small sample size, with only 25% of eligible infants providing a urine sample and only healthy newborns without clinical evidence of kidney disease, such as oedema, were included. Biological reasons for insufficient urine collections could be physiological oliguria or anuria in the first days of life due to renal immaturity in newborns (and even more so in preterm babies) [13, 14]. Furthermore, it cannot be excluded that malformations of the urinary tract and other congenital kidney diseases could have biased urine collection.

The low success rate in urine sampling could also be due to the methodological issues, e.g. insufficient urine bag adherence, newborns not urinating during the limited collection period, and no occasion for repeated collection attempts during short hospital stay of the mother and the child in uncomplicated deliveries. In addition to this initial selection bias, further study participants were lost to follow-up and persistent positive results might have been overlooked. Thus, these results should be interpreted with caution and in context.

Not surprisingly, urine collection was much more successful in boys (65.4%) than in girls (34.6%), most likely due to the better fit of urine bags to male genitals. Findings on associations between initially positive urine findings and sex and birthmode, respectively, must be interpreted with caution, since potentially confounding variables were not considered in an adjusted and longitudinal modelling design. Other variables that might be associated with initially positive urine findings, such as urinary tract infection, could not be analysed in the full sample due to missing questionnaire data, potentially introducing information bias and reducing statistical power. This needs to be considered, e.g. for power calculations, by future studies aiming for early urine testing in healthy neonates. Further, light microscopy could not be performed to verify haematuria. Due to differential urine collection success by sex and delivery mode, the reported prevalence may not represent the true prevalence in the general neonatal population. Finally, it was not possible to exclude persistent abnormalities in non-returning infants.

## Conclusions

Our findings suggest that a screening for haematuria and/or proteinuria by dipstick at birth does not provide any benefit in the early detection of kidney disease in asymptomatic neonates. If haematuria and/or proteinuria are detected in healthy infants early after birth, they need to be followed up, and they are most likely incidental or false positives. In the future, larger studies with more extensive methodology are needed to test and explore the value of urine testing for early kidney disease screening.

## Supplementary Information

Below is the link to the electronic supplementary material.

Supplementary file1 (DOCX 15 KB)

## Data Availability

The data are available upon request from the authors.

## Abbreviations

*KKHS*: KUNO Kids Health Study
*GFR*: glomerular filtration rate
*NICU*: neonatal intensive care unit
*PZN*: Pharmazentralnummer
