# Supplementary material for: Frequency and short-term persistence of haematuria and/or proteinuria in neonates: a cohort study
Source: Eur J Pediatr. 2026 Mar 26;185(4):217. doi: 10.1007/s00431-026-06859-w (PMC13021806; doi:10.1007/s00431-026-06859-w)
Supplement: Supplementary file 1 — Supplementary file1 (DOCX 15 KB) [file 431_2026_6859_MOESM1_ESM.docx]

Supplement

Table: Number of missing values in key variables in the study population with urine sample for analysis (refer exclusively to the included cohort, N=509)

| **Variable** | **Missing values (N)** |
| --- | --- |
| Sex (male/female) | 1 |
| Gestational age (weeks) | 7 |
| Birth weight (grams) | 5 |
| Delivery mode (cesarean section/vaginal) | 0 |
| Family history of kidney or urological disease (yes/no) | 67 |
| Febrile infection, sepsis or urinary tract infection within first week of life (yes/no) | 153 |
| Urinary tract infection, kidney disease or urinary tract disease within 24 weeks of life (yes/no) | 122 |

Note: The data were assessed by interview or parental questionnaires from KKHS. Family history of kidney or urological disease included kidney or urological disease of mother and father. Febrile infection, sepsis and urinary tract infection within the child’s first week of life as well as urinary tract infection, kidney disease or urinary tract disease within 24 weeks of life were aggregated into one binary variable each.
